# Metabolic Remodeling and Flavor Enhancement of Mulberry Juice Through Lactic Acid Bacteria Fermentation: A GC-IMS and Untargeted Metabolomics Approach

**DOI:** 10.3390/foods14193398

**Published:** 2025-09-30

**Authors:** Yufei Liu, Quanjun Liu, Jinglong Wang, Xianqing Huang, Yanrui Wang, Mingwu Qiao, Yan Ma, Dan Hai

**Affiliations:** 1 College of Food Science and Technology, Henan Agricultural University, Zhengzhou 450002, China; lyf11162023@126.com (Y.L.); 13523422399@163.com (Q.L.); jinglong@henau.edu.cn (J.W.); hxq8210@126.com (X.H.); 15237199474@163.com (Y.W.); mingwu0309@163.com (M.Q.); mayan201509@163.com (Y.M.); 2 Henan Engineering Technology Research Center of Food Processing and Circulation Safety Control, Zhengzhou 450002, China

**Keywords:** mulberry juice, lactic acid bacteria fermentation, flavor, metabolites

## Abstract

Fresh mulberry juice (MJ) faces industrial challenges due to its short shelf life and inconsistent flavor. This study innovatively addressed these limitations by applying *L. plantarum* (LP) and *L. fermentum* (LF) fermentation to MJ, combining non-targeted metabolomics and GC-IMS to systematically elucidate metabolic remodeling and flavor enhancement. Fermentation (36 h) achieved LAB counts > 7 log CFU/mL, significantly reducing soluble solids and pH from 15.00 to 13.90, 14.01 °Brix and 3.74 to 3.21, 3.13, respectively. In contrast, the bioactive compounds as detected by the increase in flavonoids and phenolics from 254.85 mg/100 g to 289.36, 291.39 mg/100 g and 286.21 mg/100 g to 294.55, 302.2033 mg/100 g, respectively. Anthocyanin content as high as 165.88 and 156.69 mg/L. Metabolomics identified enriched amino acid pathways, and GC-IMS revealed unique flavor profiles. The study fills a research gap by demonstrating LAB fermentation’s dual role in extending MJ’s shelf life and improving its functional nutritional quality, offering a novel strategy for functional food development.

## 1. Introduction

Mulberry, also known as mulberry fruit or mulberry berry, is a deciduous tree species belonging to the genus *Morus* of the *Moraceae* family. It is widespread throughout parts of Europe, North America and Asia, especially in provinces such as Shandong, Henan, and Sichuan in China [1,2]. Mulberry fruits are rich in various nutrients and bioactive substances, including vitamin C, vitamin E, anthocyanins, and resveratrol, which exhibit significant biological activities such as antioxidant, anti-inflammatory, anti-tumor, and immunomodulatory effects [3,4,5]. However, the thin peel and high juiciness of mulberries pose significant challenges for storage and transportation [6]. Current preservation methods include modified atmosphere packaging (e.g., low O_2_ and high CO_2_), low-temperature storage with film packaging, chemical preservatives (e.g., propionic acid, potassium sorbate), biological agents (e.g., *Bacillus subtilis*, chitosan-based coatings), and plant growth regulators (e.g., humamine, melatonin). While these approaches extend shelf life by suppressing respiration, microbial growth, or metabolic activity, they suffer from drawbacks such as chemical residues or high costs. In contrast, LAB fermentation presents a promising alternative, leveraging its natural antimicrobial metabolites and eco-friendly profile for mulberry preservation.

LAB, as one type of fermentation starter, are widely used in fruit juice fermentation. They produce organic acids such as lactic acid and acetic acid, which lower the pH and inhibit the growth of harmful microorganisms. Additionally, LAB can produce exopolysaccharides and bacteriocins, among other bioactive compounds, enhancing the health-promoting functions of the juice [7,8,9]. Numerous experiments have confirmed the effectiveness of LAB fermentation in fruit juice applications. For instance, a research team fermented blueberry juice with *L. acidophilus*, *L. plantarum*, and *L. rhamnosus*, resulting in a significant increase in phenolic content and antioxidant activity [10]. Another study found that fermentation with *L. brevis* and *L. plantarum* improved the taste of kiwifruit juice and generated new flavor compounds [11]. Furthermore, research has reported the effects of LAB fermentation on the physicochemical properties, functional characteristics, and metabolites of apple juice, orange juice, and other fruit juices [7,12,13]. Recent studies have shown that specific LAB fermentation can significantly enhance the antioxidant activity and nutritional value of mulberry juice. For example, a research team fermented mulberry juice with *L. plantarum*, resulting in a marked increase in anthocyanin content and antioxidant activity. Mouse experiments also demonstrated that fermented mulberry juice improved the cognitive abilities of aging mice by 30.3% [14].

However, research on LAB fermentation of mulberry juice (MJ) remains limited, particularly regarding the dynamic changes in flavor compounds and strain-specific metabolites during fermentation. These components critically influence the aroma, taste, and quality attributes of fermented juices. While previous studies have explored the general effects of LAB fermentation on fruit juices, there is a lack of comprehensive research on the strain-dependent metabolic signatures and flavor modulation mechanisms in mulberry juice.

Based on addressing the industrial bottleneck of the short shelf life characteristic of fresh mulberries and the targeted improvement of flavour and sensory quality. This study fills the research gap by systematically investigating the metabolic remodeling and flavor enhancement of mulberry juice through LAB fermentation using non-targeted metabolomics and GC-IMS analysis. In this study, *L. plantarum* LP69 and *L. fermentum* LF15885, isolated from fermented foods, were employed to ferment mulberry juice. Non-targeted metabolomics and GC-IMS were applied to systematically evaluate the effects of LAB fermentation on the physicochemical properties, flavor profile, and biological activity of juice. The findings aim to advance the comprehensive utilization of mulberries and their juices while highlighting the potential of LAB in functional fermented beverage production.

## 2. Materials and Methods

### 2.1. Materials and Reagents

Morus cathayana ‘Huasang No.1’ was sourced from the mulberry plantation in Shangqiu, Henan Province, China. The mulberries used were harvested in mid-May, with a sugar-to-acid ratio of 16.1 ± 0.2, soluble solids of 14.2 ± 0.4 °Brix, and a deep purple color (L = 28.4 ± 1.2; a* = 12.6 ± 0.8; b* = −2.3 ± 0.4). MRS broth, KCl, anhydrous sodium acetate, and citric acid buffer solution were obtained from Shanghai MacLean Biochemical Technology Co., Ltd., Shanghai, China; n-Octane was purchased from Shanghai Aladdin Biochemical Technology Co., Ltd., Shanghai, China; 2-octanol (analytical grade) was obtained from Shanghai YuanYe Biotechnology Co., Ltd., Shanghai, China; methanol, formic acid, and acetonitrile were supplied by Thermo Fisher Scientific, Waltham, MA, USA.

### 2.2. Strain Sources and Preparation of Fermented Mulberry Juice

*L. plantarum* LP69 (LP) and *L. fermentum* LF15885 (LF) were isolated from yogurt and a carrot–seabuckthorn juice ferment, respectively, in Henan Province, China. Both strains were stored at −80 °C. Activated cell suspensions were inoculated into pasteurized MJ (80 °C for 20 min) at optimized inoculum levels and subsequently fermented under optimized fermentation conditions (37 °C, 36 h, 3% inoculum *v*/*v*) [15]. Similarly, UFMJ was incubated under the same treatment conditions. Eventually, three distinct juice samples were obtained: unfermented mulberry juice (UFMJ), LP-fermented mulberry juice (LP), and LF-fermented mulberry juice (LF).

### 2.3. Color and Sensory Assessment

#### 2.3.1. Sensory Evaluation

A panel of 30 professionally trained assessors from Henan Agricultural University (15 males and 15 females) conducted sensory evaluations of FMJ in accordance with standardised criteria (GB/T 23776 [16] for sensory analysis and ISO 13299 [17] for sensory profiling), (Zhou et al., 2025) [15]: color (20 points; 15–20 = deep red/uniform, 6–14 = moderate, <6 = dark/variegated), taste (40 points; 30–40 = balanced, 20–29 = mild imbalance, 10–19 = excessive sweet/sour, <10 = poor harmony), aroma (20 points; 15–20 = strong mulberry, 8–14 = moderate with off-notes, <8 = pronounced off-flavors), and texture (20 points; 15–20 = uniform, 7–14 = slight precipitation, <7 = uneven). During the assessment, purified water was provided to ensure a clean palate between samples. Additionally, electronic nose and tongue technologies were employed to verify the sensory scores.

#### 2.3.2. Color Assessment

The color parameters of both fermented and unfermented samples were determined using a colorimeter (Chroma meter cr-5, Shanghai, China).

### 2.4. Determination of Physical and Chemical Parameters

For the fermentation strain, LAB in the logarithmic growth phase were used, with concentration units expressed as log CFU/mL. The pH values of MJ samples were measured using a portable pH meter (pHS-3C, THS Co., Osaka, Japan). The total soluble solids content (°Brix) was determined using an Abbe refractometer (15257, Infitek, Shanghai, China). Total acidity was determined following GB 5009.239-2016 [18], the National Food Safety Standard for the Determination of Acidity in Foods.

### 2.5. Bioactive Substances Determination

The total polyphenol content (TPC) in FMJ was measured using a modified Folin–Ciocalteu method [19]. Briefly, samples were diluted to an appropriate concentration with deionized water. An aliquot of 1 mL of the sample solution was mixed with 400 μL of Folin–Ciocalteu reagent, followed by the addition of 400 μL of 12% sodium carbonate solution. The volume was adjusted to 5 mL with distilled water, mixed, and allowed to react in the dark at room temperature for 2 h. Absorbance was then measured at 765 nm, and a standard curve was prepared using gallic acid.

The total flavonoid content (TFC) in the FMJ was determined using a slightly modified aluminum nitrate method [20]. Samples were first diluted to the appropriate concentration. Then, 140 μL of 5% (*m*/*v*) sodium nitrite solution was added, mixed, and incubated in the dark for 6 min. Following this, 140 μL of 10% aluminum nitrate solution was introduced, mixed, and incubated in the dark for another 6 min. Subsequently, 2 mL of 4% sodium hydroxide was added, and the volume was adjusted to 5 mL with 60% ethanol. The mixture was reacted in the dark at 25 °C for 15 min, and the absorbance was measured at 510 nm. A rutin standard curve was used for calibration.

The anthocyanin content of the samples was determined using a modified pH differential method according to Yan [21]. Specifically, 1 mL of the sample supernatant was diluted to 5 mL with pH 3.0 citrate buffer. Then, 1 mL of the diluted solution was further diluted to 10 mL with either pH 1.0 (KCl-HCl) or pH 4.5 (CH_3_COONa) buffer. After incubating in bath for 20 min, the absorbance was measured at 520 nm and 700 nm. Deionized water was used as a blank.

### 2.6. Volatile Compound Analysis

The volatile compounds in FMJ were analyzed by gas chromatography–ion mobility spectrometry (GC-IMS). In detail, 2 mL of the sample was accurately pipetted into a 20 mL headspace vial, along with 20 µL of 2-octanol solution (100 ppm) added as an internal standard. The vial was incubated at 40 °C under agitation at 500 rpm for 15 min. Subsequently, 200 µL of the headspace gas was injected in splitless mode into the GC-IMS system (G.A.S., Dortmund, Germany). All samples were measured in triplicate.

Chromatographic separation was carried out using an MXT-WAX capillary column (15 m × 0.53 mm, 1.0 µm; Restek, Bellefonte, PA, USA) maintained at 60 °C. High-purity nitrogen (≥99.999%) served as the carrier gas. The flow rate was programmed as follows: initial hold at 2.0 mL/min for 2 min, increased linearly to 10.0 mL/min over 8 min, then raised to 100.0 mL/min over 10 min, and held for another 10 min, resulting in a total run time of 30 min. The injector temperature was set to 80 °C.

For IMS detection, a tritium (^3^H) ionization source was employed. The drift tube was 53 mm in length and maintained at 45 °C, under an electric field strength of 500 V/cm. High-purity nitrogen (≥99.999%) was used as the drift gas at a constant flow of 75.0 mL/min. The instrument was operated in positive ion mode with a shutter opening time of 100 µs and a closing voltage of 90 dgt. Compound identification was achieved by matching against both the GC retention index database (NIST 2020, Waltham, MA, USA) and the IMS drift time database within the VOCal software (0.4.10).

### 2.7. Untargeted Metabolomics Product Analysis

Metabolite profiling of mulberry juice was analyzed using a combination of an ultra-high-performance liquid chromatography system (UltiMate 3000, Thermo) and a high-resolution mass spectrometer (Orbitrap Exploris 480, Thermo) [22]. The MJ was ultrasonically extracted (30 min, 5 °C), followed by centrifugation (4 °C, 12,000 rpm, 5 min). The supernatant was then vacuum-centrifugally concentrated for 4 h, and after adding methanol-water, it was sonicated and filtered prior to LC-MS analysis. Chromatographic separation was performed on an HSS T3 column at 50 °C with a flow rate of 0.4 mL/min. LC-MS analysis was conducted in both positive and negative ion modes, scanning from 67 to 1000 *m*/*z*. The ion spray voltage was set to 3500 V (positive) and 2500 V (negative). The sheath gas (Arb) was set to 50, auxiliary gas (Arb) to 10, and sweep gas (Arb) to 1. The ion transfer tube temperature was maintained at 325 °C, and the vaporizer temperature at 350 °C, with collision energy varying from 20 to 60 V. The resolution was set to 30,000, and data were collected in data-dependent acquisition (DDA) mode. The raw data were analyzed with Compound Discoverer (Thermo Scientific, USA) to perform baseline filtering, peak recognition, integration, retention time adjustment, and peak alignment. This processing generated a data matrix containing retention time, mass-to-charge ratio, and peak intensity. The primary databases used included https://www.mzcloud.org/, (accessed on 26 February 2025), http://www.hmdb.ca/ (accessed on 26 February 2025), and other Thermo databases, public databases, and in-house databases. The final data matrix was obtained for subsequent analysis.

### 2.8. Data Statistical Analysis

The experiment was conducted with three biological replicates. The statistical analysis was performed using SPSS 27.0 (IBM, Armonk, NY, USA), with quantitative data expressed as mean ± standard deviation. For multiple group comparisons, one-way ANOVA followed by Tukey’s post hoc test was conducted, with statistical significance set at *p* < 0.05. Prior to analysis, the normality of data distribution was assessed using the Kolmogorov–Smirnov test, while homogeneity of variance was verified by Levene’s test. Graphical representations were generated using Origin 2021 software (OriginLab, Northampton, MA, USA).

## 3. Results and Discussion

### 3.1. Identification of Fermentation Strains

**Figure 1:**
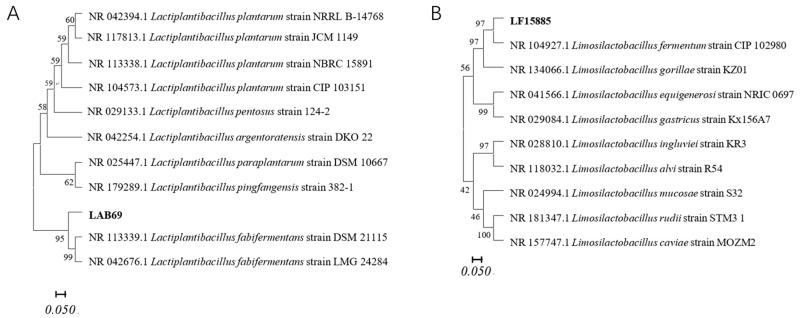
Genotypes of target fermentation strains: *Lactobacillus plantarum* (**A**); *Lactobacillus fermentum* (**B**).

Our preliminary work has isolated and screened target fermentation strains, which were subsequently identified and subjected to 16S rDNA analysis. As clearly demonstrated in Figure 1A,B, LP corresponds to *Lactobacillus plantarum*, while LF corresponds to *L. fermentum*.

### 3.2. FMJ’s Color Difference and Sensory Evaluation

**Figure 2:**
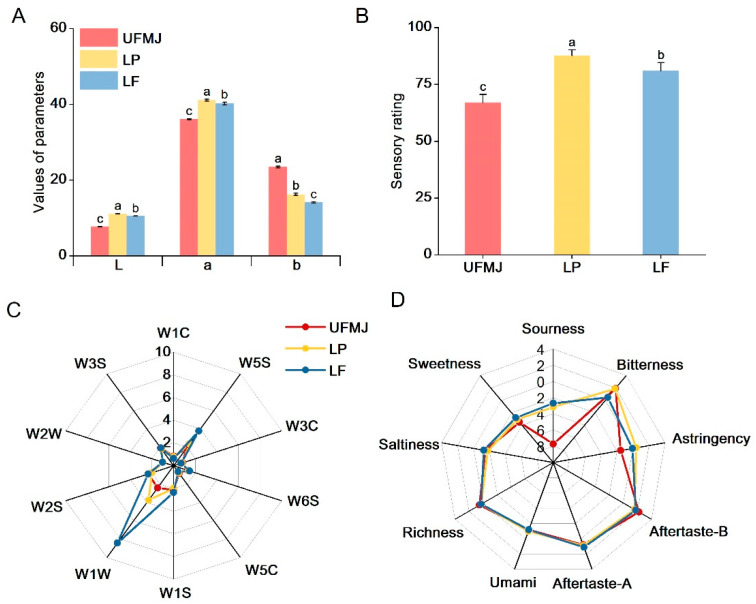
Changes in physical, chemical and sensory characteristics of fermented mulberry juice. (**A**) Values of parameters (L*, b*, c*), (**B**) Sensory rating, (**C**) Electronic nose, (**D**) Electronic tongue. Letters a–c indicate statistically significant differences (*p* < 0.05).

Color plays a crucial role in food, affecting not only the sensory quality but also the consumer acceptance directly [23]. In this study, the color characteristics of UFNJ and FMJ were characterized using color parameters (L*, a*, and b*, Figure 2A). Fermentation with LP and LF strains significantly increased the L* and a* values of the juice, while the b* value decreased (*p* < 0.05). This indicates that LAB fermentation enhances the brightness and red hue of MJ, reducing the yellow chroma, consistent with results from LAB fermentation of cherry juice [15]. Additionally, we observed a close correlation between the color characteristic changes in FMJ and the pH value during fermentation (*p* < 0.05). Studies indicate that anthocyanins predominantly accumulate as flavylium cations under low pH conditions, thereby enhancing the red color intensity of fruit juices [24].

**Figure 3:**
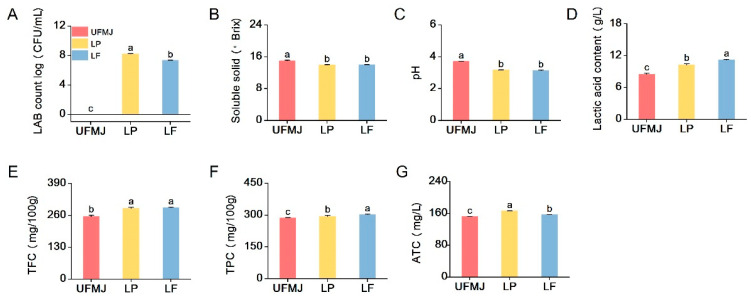
Changes in the count of chemical and functional substances of fermented mulberry juice. (**A**) The number of live LAB, (**B**) Soluble solids, (**C**) pH, (**D**) Lactic content, (**E**) TFC, total flavonoid content, (**F**) TPC, total polyphenol content, (**G**) ATC, anthocyanins. Letters a–c indicate statistically significant differences (*p* < 0.05).

In this study, the pH of FMJ significantly decreased to below 3.20 (*p* < 0.05) (Figure 3C). The reduced pH and enhanced juice brightness suggest that the increased redness of FMJ may be associated with pH reduction. Collectively, fermentation markedly altered the color characteristics of mulberry juice, resulting in brighter and redder appearance. These changes highlight the impact of LAB fermentation on MJ color quality, potentially enhancing its consumer appeal [25].

Compared to UFMJ, we further systematically evaluated the overall sensory acceptability of FMJ (Figure 2B). FMJ exhibited significantly higher sensory scores than UFMJ (*p* < 0.05). The sensory score of FMJ was significantly higher than that of UFMJ (*p* < 0.05). Radar charts from electronic nose and electronic tongue analysis are shown in Figure 2C,D. As seen in Figure 2C, significant differences in the response intensity of different groups of juices to the sensors were observed, with FMJ showing significantly higher responses for W1W (sulfides) and W5S (carbon oxides) compared to UFMJ (*p* < 0.05). Meanwhile, as shown in Figure 2D, UFMJ had stronger richness, bitterness, and aftertaste, while FMJ showed stronger responses to sourness, umami, and astringency (*p* < 0.05). This is similar to Mandha’s study, where UFMJ had stronger richness, bitterness, and aftertaste, while LAB-fermented juice showed stronger responses to sourness, saltiness, and umami [26].

### 3.3. Physicochemical Properties of FMJ

The total colony count of LAB directly reflects the adaptability of LAB in the mulberry juice matrix (*p* < 0.05). During the 37 °C, 36 h fermentation process, the total LAB colony count of LP and LF strains increased from 1.03 ± 0.04 and 0.87 ± 0.56 CFU/mL to 8.21 ± 0.04 and 7.34 ± 0.15 CFU/mL (Figure 3A), indicating strong adaptability and proliferation ability of the two strains in the juice. FMJ is a suitable substrate for fermentation and growth of the two LAB strains in this study. In contrast, no viable bacteria were detected in UFMJ at 0 and 36 h of fermentation. Changes in soluble solids and acidity further reflect the adaptability and proliferation ability of LAB strains in MJ. Soluble solids are particularly important as the main source of carbon during LAB fermentation. As shown in Figure 3B, the soluble solids in mulberry juice fermented by the two LAB strains decreased to 13.9 ± 0.1 and 14.01 ± 0.01, respectively, compared to UFMJ (15 ± 0.16) (*p* < 0.05). Correspondingly, the pH value also decreased. The pH values of both LP and LF fermented FMJs were significantly lower than that of UFMJ (pH = 3.74 ± 0.01). Among them, the pH value of LF fermented mulberry juice was the lowest (3.13 ± 0.02), indicating that the LF strain fully utilized the carbon source in the juice for fermentation and had the strongest acid production capacity, which is highly consistent with the lactic acid content results in Figure 3C. These changes are due to the consumption of sugar by LAB during fermentation, producing a large amount of organic acids.

Previous research has demonstrated that *Lactobacillus plantarum* utilizes hexoses—including fructose, mannitol, and galactose—which are converted into pyruvate via glycolysis; pyruvate is subsequently reduced to lactic acid by lactate dehydrogenase, leading to an elevation in lactic acid concentration [27]. This is consistent with our results. A study also showed that apple juice fermented by *Lactobacillus plantarum* had a pH value decrease from 3.70 to 3.40 after 50 h of fermentation [13]. The variation in soluble solids and lactic acid content among different strains could be attributed to disparities in how LAB utilize and biotransform sugars within the fermentation substrate [28]. In summary, different LAB strains have a significant impact on the fermentation characteristics of mulberry juice.

### 3.4. Analysis of Bioactive Components in FMJ

Phenolics, flavonoids, and anthocyanins have been recognized for their beneficial biological activities and play a significant role in maintaining human health and preventing diseases [29,30]. As illustrated in Figure 3E–G, the contents of total flavonoids, total polyphenols, and anthocyanins in FMJ were significantly higher than those in UFMJ. Compared to UFMJ, TFC rose by 13.78% (289.36 ± 4.28 mg/100 g) in LP and 14.57% (291.39 ± 2.53 mg/100 g) in LF. TPC increased by 2.91% (LP) and 5.56% (LF), while TAC reached 165.88 ± 0.48 mg/100 g (LP) and 156.69 ± 0.51 mg/100 g (LF). These changes align with previous research findings [31,32]. The increase in TPC and TFC in MJ after fermentation may be attributed to the ability of LAB hydrolases to break down plant cell walls, releasing polyphenols and flavonoids that were originally bound to the cell walls [33]. Furthermore, LAB fermentation can promote the hydrolysis of anthocyanin glycosides, releasing more stable anthocyanidins and metabolic byproducts such as aldehydes, acetic acid, and lactic acid, which interact to slow down degradation and maintain the deep red color of FMJ [34,35].

### 3.5. Changes in Volatile Compounds

**Figure 4:**
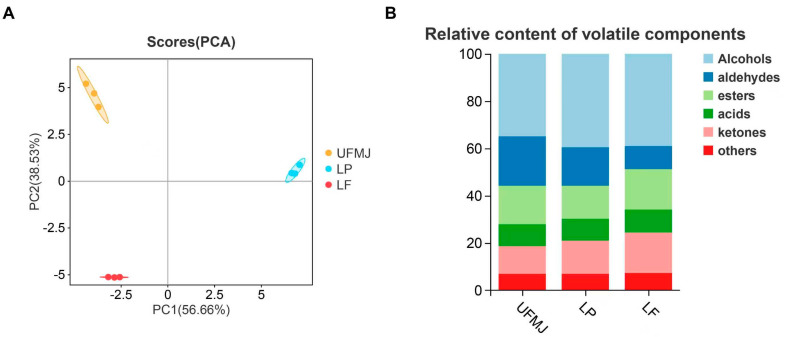

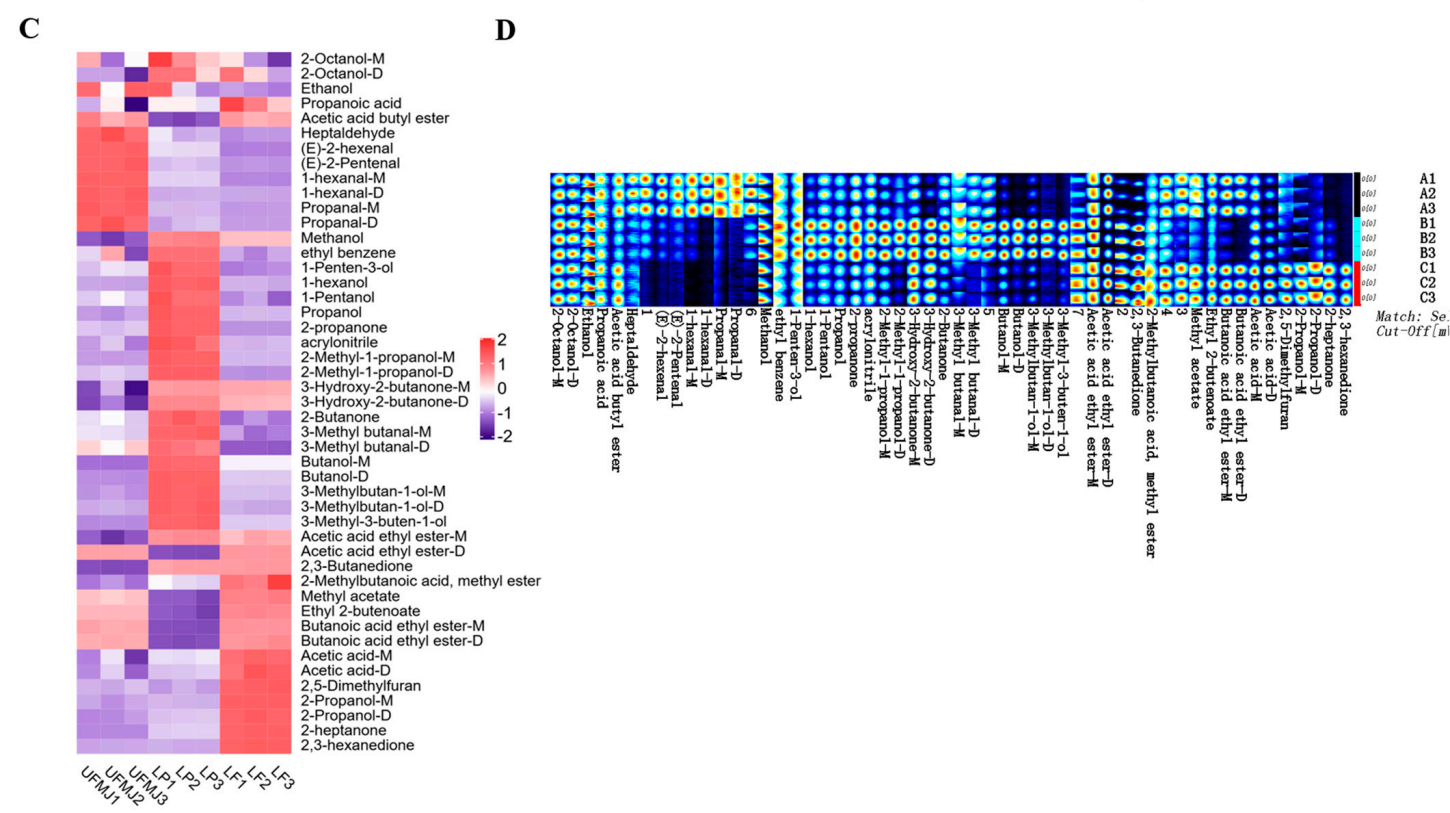
Changes in flavor of fermented mulberry juice. (**A**) PLS-DA plots, (**B**) Classification and analysis of metabolites, (**C**) Hierarchical clustering heatmap (*p* < 0.05 and VIP > 1), (**D**) Finger pattern. Representative samples showing the comparative analysis of (A1–A3) unfermented mulberry juice (UFMJ), (B1–B3) Lactobacillus plantarum-fermented MJ (LP), and (C1–C3) Lactobacillus fermentum-fermented MJ (LF).

Volatile substances are key factors influencing the flavor, aroma, and consumer acceptance of fermented fruit juices. Analysis of volatile compounds in FMJ using GC-IMS identified a total of 54 chemical substances, categorized into acids (6 types), esters (5 types), alcohols (17 types), aldehydes (9 types), ketones (7 types), and others (3 types) (Figure 4B). Among these, alcohols and aldehydes are the primary volatile components, contributing to the fruity, fresh, and full-bodied mouthfeel of the fermented juice. In the Principal Component Analysis (PCA) plot, clear separation was observed between the two types of FMJ and UMFJ, suggesting that fermentation markedly changed the flavor profile of MJ (Figure 4A). A heatmap of 47 highly abundant metabolites further revealed that LAB fermentation elevated the concentrations of certain volatile flavor substances, contributing to an improved flavor of MJ (Figure 4C). To better understand how these changes in volatile compounds influence the flavor attributes of MJ and interact to shape the overall flavor profile, the specific contributions of key compounds were further analyzed. Acetic acid ethyl ester-M exhibited a strong fruity aroma, commonly found in bananas, strawberries, and melons, and its enrichment after LAB fermentation enhanced the fruity and sweet notes of FMJ [10]. 2,3-Butanedione and 3-Hydroxy-2-butanone, known for their mild creamy aromas, were also significantly enriched, and their ratio could indicate the completeness of fermentation [36]. These three compounds were markedly enriched in both types of FMJ. Conversely, fermentation reduced the concentrations of certain compounds that adversely affect flavor, such as propanal, 1-hexanal, and (E)-2-pentenal, which have intense spicy and grassy notes. These changes were consistent with previous findings [37].

In conclusion, LAB fermentation markedly altered the concentration and profile of volatile compounds in mulberry juice, improving its overall aroma and taste through the accumulation of desirable constituents and the reduction in less favorable ones. The fermentation outcomes varied among different LAB strains, highlighting their distinct metabolic capabilities. For example, the LF strain promoted the accumulation of acids and alcohols, compounds recognized for enhancing the acidic and aromatic sensory attributes of fruit products [21]. By contrast, the impact of the LP strain was more pronounced on alcohols and aldehydes, which are typically responsible for generating the vinous, sweet, and characteristic fruity flavor notes in fermented juices [32,38]. The modifications in flavor compounds were consistent with the sensory analysis outcomes of FMJ, underscoring the critical role of strain-dependent metabolic activities in defining the ultimate flavor characteristics. These findings stress the interplay between bacterial fermentation and volatile compound dynamics, as well as their combined contribution to the overall flavor, offering key insights for refining fermentation strategies to attain targeted sensory qualities.

### 3.6. Metabolic Analysis of FMJ

**Figure 5:**
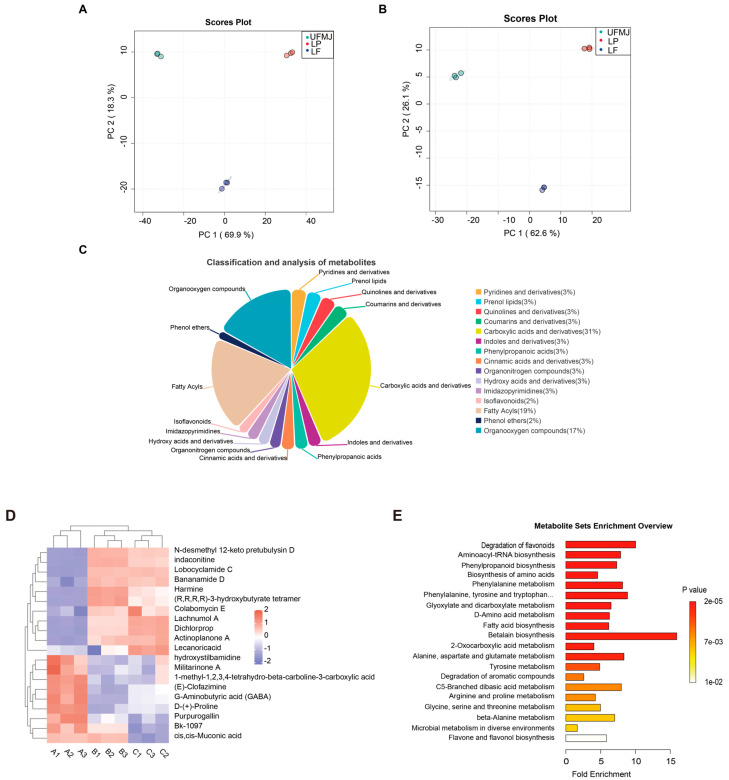
Effects of Lactobacillus fermentation on the metabolic profile of mulberry juice. (**A**) PCA plot of positive ion mode (**B**) PCA plot of negative ion mode, (**C**) Relative content of volatile components, (**D**) Hierarchical clustering heatmap of differential metabolites, (**E**) KEGG functional pathway map.

We utilized untargeted metabolomics to detect changes in metabolites within FMJ. A total of 1660 metabolites were detected, with 1120 identified in positive ion mode and 540 in negative ion mode. Principal component analysis (PCA) was performed to evaluate metabolic variations induced by fermentation. Under ESI+ conditions, PC1 and PC2 explained 69.9% and 18.3% of the total variance, respectively (Figure 4A); under ESI- conditions, the corresponding values were 62.6% and 26.1% (Figure 5B). The three biological replicates within each group clustered tightly, and a distinct separation was observed between the fermented and unfermented juice samples. The results indicated significant differences in metabolites of MJ before and after fermentation. Based on the Human Metabolome Database, the identified metabolites were classified into carboxylic acids and their derivatives (30.58%), lipids and lipid-like molecules (19.42%), organic oxygen compounds (16.91%), coumarins and derivatives (3.24%), prenol lipids (3.24%), and quinolines and derivatives (3.24%), among others (Figure 5C). Organic acids and their derivatives were the most abundant in composition and content, playing a crucial role in the flavor and preservation of fruits and fermented juices [39]. The top 20 differentially abundant metabolites were selected for further cluster analysis (Figure 5D), systematically revealing the dynamic changes and biological significance of metabolites during the fermentation of mulberry juice.

The 20 metabolites exhibiting the highest differential abundance were selected for further cluster analysis (Figure 5D), systematically revealing the dynamic changes in metabolites during mulberry juice fermentation and their biological significance. Heatmap visualisation displayed distinct metabolic patterns between fermented and unfermented groups, with the terpenoids N-desmethyl 12-keto pretubulysin D and Lachnumol A significantly upregulated in the LP and LF groups. These findings are particularly noteworthy as these compounds have been experimentally demonstrated to inhibit pro-inflammatory cytokines (TNF-α, IL-6) in macrophage models and exhibit radical scavenging activity in DPPH assays, as documented in prior studies [40,41]. The observed downregulation of hydroxystilbamidine and GABA aligns with established microbial metabolic pathways, wherein LAB fermentation typically produces benzopyranone derivatives via glycosidase activity and regulates neurotransmitter precursors through glutamate decarboxylase pathways [42,43]. Harmine, a β-carboline alkaloid exhibiting moderate upregulation (≈0.4), possesses monoamine oxidase (MAO) inhibitory activity linked to antidepressant effects in rodent models [44,45]. The enrichment of Bananamide D and Harmine is consistent with their reported mechanisms of action in neurological systems, including MAO inhibition and modulation of dopamine receptors, as demonstrated in both in vitro and animal studies [44,46,47]. The ketone body polymer (R,R,R,R)-3-hydroxybutyrate tetramer displayed the most pronounced fermentation-induced elevation (>0.9), which is particularly noteworthy given its ability to cross the blood–brain barrier and serve as an alternative energy substrate during metabolic stress [48].

While Purpurogallin showed reduced levels, this change should be interpreted in the context of the overall antioxidant system, where multiple phenolic compounds may compensate through synergistic effects, as observed in other fermented fruit matrices [49]. The collective metabolic shifts suggest that LAB fermentation induces a coordinated remodeling of mulberry juice composition, favoring compounds with documented bioactive properties while maintaining functional balance through complementary metabolic pathways. These metabolic shifts collectively illustrate how LAB fermentation transforms mulberry juice into a functionally enriched beverage, with the heatmap patterns providing visual validation of pathway activation through microbial metabolism.

### 3.7. Analysis of Key Functional Metabolites

**Figure 6:**
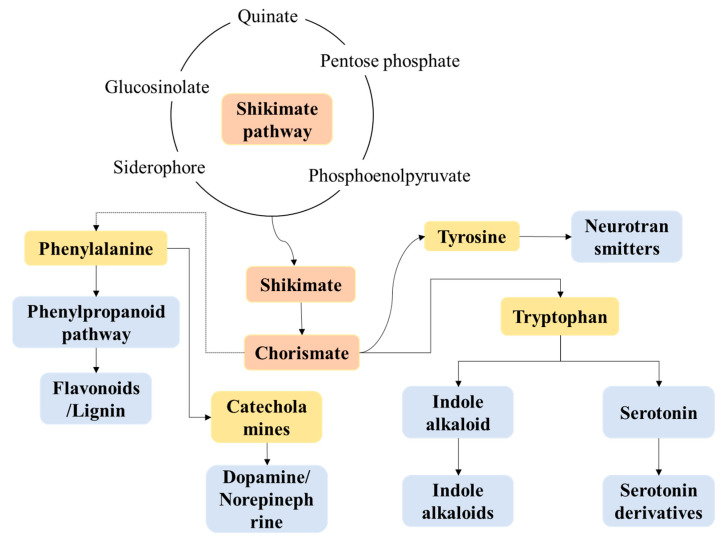
Important functional substances in the metabolism of phenylalanine, tyrosine and tryptophan.

This study utilized the KEGG metabolic pathway map (Figure 6) to delve into the metabolic networks and health effects of key metabolites such as phenylalanine, tyrosine, tryptophan, and their functional metabolites in fermented fruit juices. As illustrated in the figure, during the fermentation of mulberries by lactic acid bacteria, the shikimate pathway acts as a central hub connecting sugar metabolism to aromatic amino acid synthesis, crucially shaping the beverage’s functional properties and quality. Its carbon skeletons derive mainly from pentose phosphate pathway intermediates (supplying NADPH and 5-phosphoribose), quinic acid, and 6-oxo-sugars. These precursors support aromatic amino acid biosynthesis and influence the final product’s flavor and functionality through metabolic flux reprogramming. In aromatic amino acid synthesis, the phenylalanine branch yields flavonoids and lignin precursors via the phenylpropanoid pathway [50]. These compounds are transformed by lactic acid bacteria into antioxidants (e.g., p-coumaric and caffeic acids), which could contribute to the beverage’s free radical scavenging capacity and oxidative stability [51]. Tyrosine branches are precursors of catecholamines, which could potentially regulate vascular tension, reduce the risk of hypertension and provide potential neuroregulatory effects, and promote the formation of melanoidins through the Maillard reaction, improving color stability and aroma [28]. The tryptophan branch generates 5-hydroxytryptophan, a serotonin precursor that may convert into vasodilatory compounds, while accumulated indole alkaloids impart bitterness and aroma, defining the beverage’s “health-promoting flavor” [52,53,54].

Shikimate pathway intermediates (e.g., chorismic and anthranilic acids) produce aromatic volatiles like phenylacetaldehyde and phenylethanol, contributing to fruity and floral notes [55,56]. Tryptophan-derived indole alkaloids synergize with phenolic compounds to enhance antimicrobial activity, extending shelf life [57]. Tyrosine-derived melanin precursors protect against photooxidation. Fermentation-induced bioconversion of aromatic amino acids may yield vasodilatory bioamines, and tryptophan metabolites (e.g., serotonin) offer mood-regulating potential, aligning with functional food trends [58,59]. Furthermore, during fermentation, chorismate also contributes to the enrichment of flavonoids, which potentially suppress NF-κB signaling to reduce inflammatory cytokines, demonstrating potent antioxidant and anti-inflammatory effects [34,60]. Additionally, the tryptophan-kynurenine pathway and phenylalanine-tyrosine axis might modulate hepatic enzyme activity and autophagy, thereby potentially impacting drug metabolism and lipid homeostasis [61,62].

In summary, the proliferation of LAB alters the metabolic pathways of fermented fruit juice, enhancing the flavor, antioxidant, antibacterial, physiological regulatory, and functional properties of mulberry fermented beverages. This provides valuable insights and support for the development of novel functional fermented products.

This study elucidates the metabolic remodeling and flavor enhancement of mulberry juice (MJ) through lactic acid bacteria (LAB) fermentation, yet several limitations warrant consideration. The exclusive use of *L. plantarum* LP69 and *L. fermentum* LF15885 restricts extrapolation to other LAB strains with divergent metabolic profiles, while fixed fermentation parameters (36 h, 37 °C) may not reflect outcomes under variable conditions. This research remains at the laboratory stage; subsequent industrial collaboration requires sterilisation process optimisation and pilot-scale upscaling experiments to determine specific effects for industrial production. Small-scale laboratory fermentations may not account for industrial-scale challenges like batch consistency and scalability. Although GC-IMS and untargeted metabolomics provided comprehensive analyses, low-abundance metabolites or strain-specific interactions might remain undetected. Future research should expand to multi-strain fermentations, industrial-scale validation, and longitudinal shelf-life assessments, particularly focusing on probiotic viability via microencapsulation. Additionally, clinical trials are needed to substantiate health claims. Despite these constraints, this work establishes a critical foundation for developing functional fermented MJ, with implications for optimizing strain-specific protocols to maximize flavor and nutrient retention.

## 4. Conclusions

This study systematically investigated the metabolic remodeling and flavor enhancement of mulberry juice (MJ) through fermentation with *L. plantarum* LP69 and *L. fermentum* LF15885 using a multi-omics approach combining GC-IMS and untargeted metabolomics. LAB fermentation effectively addressed the inherent perishability of mulberry fruits by reducing pH (to ≤3.2) and soluble solids (7.3–13.3%), while significantly enhancing nutritional and sensory profiles. Strain-specific metabolic pathways (e.g., shikimate-mediated aromatic amino acid biosynthesis) drove the bioconversion of bound phenolics into bioactive forms, increasing total flavonoids by 13.8–14.6% and anthocyanins to 156.7–165.9 mg/100 g. GC-IMS analysis revealed enriched desirable volatiles (e.g., acetic acid ethyl ester) and reduced off-flavors, improving sensory scores by 30–40%. Metabolomics identified fermentation-induced metabolites (e.g., N-desmethyl12-ketopretubulysin D) with potential neuroprotective and anti-inflammatory activities [63]. These findings demonstrate LAB-fermented MJ as a commercially viable probiotic-rich functional beverage, aligning with circular economy principles by valorizing perishable raw materials without chemical preservatives. Future research should optimize strain-specific protocols and validate health claims through clinical trials.

## Data Availability

All data included in this study are available upon request by contacting the corresponding author.
